# The effect of the TIM program (Transfer ICU Medication reconciliation) on medication transfer errors in two Dutch intensive care units: design of a prospective 8-month observational study with a before and after period

**DOI:** 10.1186/s12913-017-2065-y

**Published:** 2017-02-10

**Authors:** Bertha Elizabeth Bosma, Edmé Meuwese, Siok Swan Tan, Jasper van Bommel, Piet Herman Gerard Jan Melief, Nicole Geertruida Maria Hunfeld, Patricia Maria Lucia Adriana van den Bemt

**Affiliations:** 1 Department of Hospital Pharmacy, Haga Teaching Hospital, Els Borst-Eilersplein 275, 2545 CH The Hague, The Netherlands; 2 Apotheek Haagse Ziekenhuizen (AHZ), Charlotte Jacobslaan 70, 2545 AB The Hague, The Netherlands; 3 Department of Hospital Pharmacy, Erasmus University Medical Center, PO Box 2040, 3000 CA Rotterdam, The Netherlands; 4 Institute for Medical Technology Assessment, Erasmus University Rotterdam, PO Box 1738, 3000 DR Rotterdam, The Netherlands; 5 Intensive Care Unit, Erasmus University Medical Center, PO Box 2040, 3000 CA Rotterdam, The Netherlands; 6 Intensive Care Unit, Haga Teaching Hospital, Leyweg 275, 2545 CH The Hague, The Netherlands

**Keywords:** Medication reconciliation, Medication error, Intensive care unit, Medication transfer, Clinical pharmacist, Adverse drug event

## Abstract

**Background:**

The transfer of patients to and from the Intensive Care Unit (ICU) is prone to medication errors. The aim of the present study is to determine whether the number of medication errors at ICU admission and discharge and the associated potential harm and costs are reduced by using the Transfer ICU and Medication reconciliation (TIM) program.

**Methods:**

This prospective 8-month observational study with a pre- and post-design will assess the effects of the TIM program compared with usual care in two Dutch hospitals. Patients will be included if they are using at least one drug before hospital admission and will stay in the ICU for at least 24 h. They are excluded if they are transferred to another hospital, admitted and discharged in the same weekend or unable to communicate in Dutch or English.

In the TIM program, a clinical pharmacist reconciles patient’s medication history within 24 h after ICU admission, resulting in a “best possible” medication history and presents it to the ICU doctor. At ICU discharge the clinical pharmacist reconciles the prescribed ICU medication and the medication history with the ICU doctor, resulting in an ICU discharge medication list with medication prescription recommendations for the general ward doctor. Primary outcome measures are the proportions of patients with one or more medication transfer errors 24 h after ICU admission and 24 h after ICU discharge. Secondary outcome measures are the proportion of patients with potential adverse drug events, the severity of potential adverse drug events and the associated costs.

For the primary outcome relative risks and 95% confidence intervals will be calculated.

**Discussion:**

Strengths of this study are the tailor-made design of the TIM program and two participating hospitals. This study also has some limitations: A potential selection bias since this program is not performed during the weekends, collecting of *potential* rather than *actual* adverse drug events and finally a relatively short study period.

Nevertheless, the findings of this study will provide valuable information on a crucial safety intervention in the ICU.

**Trial registration:**

Dutch trial register: NTR4159, 5 September 2013

## Background

Patients in the intensive care unit (ICU) are more likely to experience medication errors than other hospitalized patients, since the chance of errors in an ICU setting is high due to the high intensity of treatment in patients with a critical illness [1, 2]. Medication errors can induce adverse events and are associated with substantial costs [3–10]. It has been demonstrated that chronic medication used by patients before admission to the ICU is often unintentionally discontinued after the ICU stay [11]. Because of the critical illness, non-vital medication is often temporarily withheld. However when the patient improves, the restart of this medication is easily forgotten. On the other hand, drug therapy initiated for short-term use in the ICU can be inadvertently continued after ICU discharge. For example, several studies have demonstrated that gastric acid-inhibitory drugs are frequently continued inappropriately after discharge from the ICU [12–14].

On regular wards, many Dutch hospitals use computerized physician order entry systems with clinical decision support (CPOE/CDS). In general, these CPOE/CDS systems cannot offer supporting systems for patient monitoring required at ICUs. Therefore most ICUs in the Netherlands do not use the general CPOE/CDS system. Instead, they use a so-called Patient Data Monitoring System (PDMS). However, these systems mostly lack clinical decision support for prescribing and monitoring medication. Besides, the use of two different systems poses an additional threat to safe medication transfer to and from the ICU.

Various interventions have been studied to reduce medication errors on the ICU. A systematic review by Manias et al. identified eight types of interventions: implementation of CPOE, changes in working schedules, intravenous systems, modes of education, medication reconciliation, pharmacist involvement, protocols an guidelines and support systems for clinical decision making. Only four of these interventions demonstrated reduced medication errors post-intervention, one of which was medication reconciliation at ICU admission [15]. Besides, a small number of studies suggest that that the incidence of medication errors during and after hospitalization can be reduced even further by additional medication reconciliation at ICU discharge [16–18].

However, the effect of medication reconciliation after ICU stay is not well described in the literature. The few available studies suffer from one or more of the following limitations: (1) small sample size; (2) failure to differentiate between intentional and unintentional discrepancies and (3) lack of assessment of potential clinical impact and/or severity of discrepancies. Besides, it is not known how patient safety will be affected by a combined admission and discharge medication reconciliation program in the ICU.

In the present study, we hypothesize that continuity of care will be improved and medication errors and patient harm will be reduced if medication is reconciled during both ICU transfers by a pharmacist in close collaboration with the intensive care doctors and the ICU staff. To test this hypothesis, we designed the TIM (*T*ransfer *I*CU and *M*edication reconciliation) program, which consists of medication reconciliation by a pharmacist at ICU admission and at ICU discharge.

## Methods/Design

### Aim of the study

The aim of this study is to determine the effect of the TIM program on the number of medication transfer errors (MTEs) at admission to and at discharge from the ICU. Furthermore, we will investigate the effect of the program on the number of potential adverse drug events and their severity, and finally we will estimate the cost-effectiveness of this program.

### Design

A prospective 8-month observational study with a before and after design will be carried out at Haga Teaching Hospital in The Hague (650 bed general teaching hospital with 18 ICU beds; 1500 ICU admissions a year) and Erasmus Medical Center in Rotterdam (University Hospital, 1320 beds and 32 ICU beds; 1800 ICU admissions a year). Both hospitals are located in the Netherlands.

**Fig. 1:**
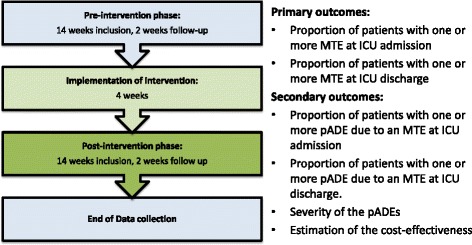
Study flow chart of the TIM program and defined outcomes. ICU = intensive care unit, MTE = medication transfer error, pADE = *potential* adverse drug event, TIM = Transfer ICU and Medication reconciliation program

The study consists of three periods: first, a pre-intervention period of 14 weeks during which patients will be included who receive usual care; second, a period of 4 weeks during which the TIM intervention program will be implemented, and finally, a post intervention period of 14 weeks during which patients will be included in the TIM program (Fig. 1).

### Study population

All patients admitted to the ICU during the study period are eligible to participate in this study if they use at least one drug at home and if the ICU length of stay exceeds 24 h. An ICU discharge and readmission within 24 h is counted as one inclusion. Patients who die during the ICU stay are included in the admission part of the study. Readmissions to the ICU are also included.

Exclusion criteria are transfer to another hospital, both admission and discharge within the same weekend (Friday 17:00 until Monday 8:30) and patient’s inability to be counseled in Dutch or English.

### Study procedures

#### Usual care at ICU admission (pre-intervention period)

At ICU admission the ICU doctors determine pre-admission medication. In this process, they rely on the data from the previous ward (if applicable), the patient’s general practitioner, relatives or previous hospital records (e.g. discharge letters, patient charts). Occasionally, a faxed medication list is available from the community pharmacy, but there is no standard procedure for the ICU doctor to obtain medication lists from the community pharmacy. Hence, ICU doctors do not structurally perform medication reconciliation. The admitting doctor registers pre-admission medication in the PDMS system (Haga) or HIS (Hospital Information System) (Erasmus MC) and only transcribes the medication that is to be continued during the ICU stay into the PDMS/CPOE

In Erasmus MC a dedicated ICU hospital pharmacist reviews the prescribed medication on the ICU on a daily basis during patient rounds. In Haga Teaching Hospital, twice a week, one out of a team of 4 trained ICU hospital pharmacists reviews prescribed medication and attends the patient rounds on the ICU. In both hospitals, the hospital pharmacists rely on the information about pre-admission medication available in the PDMS and do not obtain a medication list from the community pharmacy, nor do they reconcile pre-admission medication with the patient.

#### Usual care at ICU discharge

An ICU doctor writes an ICU discharge letter, which is screened, verified and authorized by an intensivist. Combined with a nurse discharge letter, this discharge letter is sent to the admitting ward at the moment of patient transfer.

In Haga Teaching Hospital, the discharge letter contains information about the registered pre-admission medication, the medication in use at discharge from the ICU and suggestions about medication use after discharge (i.e.: “Restart pre-admission medication”). In Erasmus MC, the letter contains information about medication in use at discharge and sometimes suggestions for medication use after discharge.

After transfer, the doctor of the admitting ward has to transcribe medication orders from the discharge letter to the hospital CPOE/CDS. At the same time, doctors in the Haga Teaching Hospital have to stop medication registered in the CPOE/CDS that the patient is no longer required to take after ICU discharge, since the CPOE/CDS system in this hospital does not automatically stop prescribed medication when the patient is admitted to the ICU. By contrast, the CPOE/CDS system in Erasmus MC stops all medication at admission to the ICU, which urges the doctor to prescribe all medication needed after the ICU transfer.

#### TIM program, the intervention

The TIM program consists of medication reconciliation at ICU admission and at ICU discharge. In both intervention hospitals, the program will be carried out by two pharmacists. The pharmacists in Erasmus MC will be supported by pharmacy students, who, after a training period of several weeks, will be able to collect the medication lists and to interview the patients or their relatives about each patient’s pre-admission medication.

#### TIM program at ICU admission

First, permission for reconciliation is obtained from the patients or their relatives. Next, the pharmacist compiles a best possible medication history (BPMH) as described below in the section “the gold standard medication lists”. In case of an internal transfer, the BPMH will also include the pre-admission medication transcribed or omitted at the previous ward.

Next, the pharmacist transfers the BPMH to the PDMS system (Haga) or HIS (Erasmus MC) and presents the BPMH to the ICU doctor responsible for the patient. If the pharmacist notices an inexplicable discrepancy between pre-admission medication and medication prescribed on the ICU, he or she will discuss this with the ICU doctor, enabling correction of unintended omissions or other mistakes in the ICU prescriptions. Finally, the pharmacist in Haga Teaching Hospital stops the patient’s medication in the CPOE/CDS system used on the wards, which is done automatically in the HIS system of Erasmus MC when the patient is admitted to the ICU.

#### TIM program at ICU discharge

**Fig. 2:**
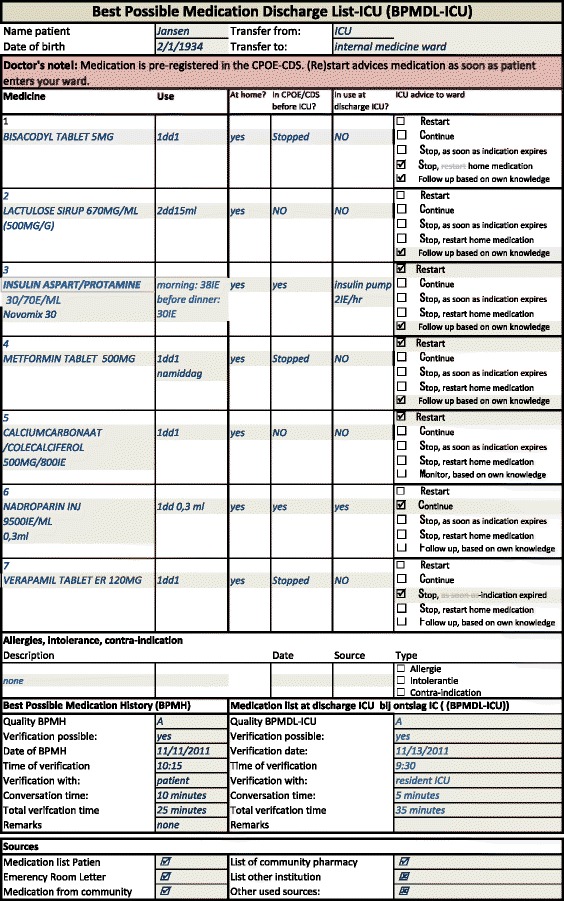
Example of BPMDL-ICU. BPMDL-ICU = best possible ICU medication discharge list. N.B. This is a fictitious example, patient and doctor names are imaginary

When a patient is discharged from the ICU, the pharmacist makes a discharge medication summary. This medication summary contains an overview of medications on the patient’s BPMH combined with all the medication the patient uses in the ICU at the moment of discharge. For each drug, the ICU doctor is given a series of options of recommendations (i.e.: restart, stop, continue), making it easier for the ICU doctor to provide clear prescription suggestions to the doctor of the general ward. The ICU pharmacist reconciles this medication list with the ICU doctor. During this reconciliation, special attention is paid to temporarily withheld medication that should be restarted and to temporary ICU medication that should be stopped. After this reconciliation, the pharmacist amends the medication list to establish the Best Possible ICU Medication Discharge List (BPMDL-ICU) (example in Fig. 2). Once the doctor has approved the BPMDL-ICU, it is combined with the discharge letter of the ICU and sent to the doctor of the regular ward at the moment of patient transfer.

In Erasmus MC, the pharmacist pre-registers the BPMDL-ICU in the CPOE/CDS system, so the ward doctor merely has to authorize this pre-registered medication list. Since pre-registration is not possible in the CPOE/CDS system of Haga Teaching Hospital, here the medication on admission is registered in the outpatient CPOE/CDS system, from where the ward doctor can easily transcribe this medication list to the inpatient CPOE/CDS system.

**Fig. 3:**
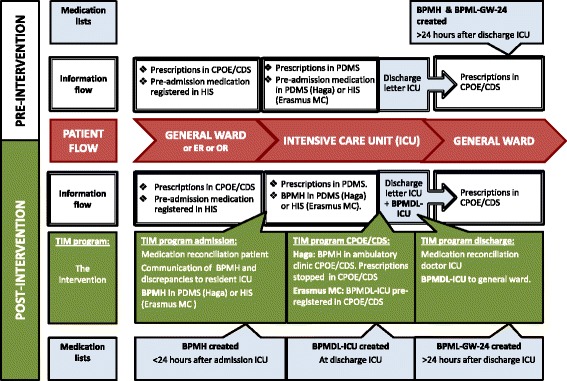
Study procedure pre- and post-intervention. BPMDL-ICU = best possible ICU medication discharge list, BPMH = best possible medication history, BPML-GW24 = best possible general ward medication list 24 h after ICU discharge, CPOE/CDS system = computerized physician order entry systems with clinical decision support, ER = emergency room, HIS = hospital information system, ICU = intensive care unit, OR = operating room, PDMS = patient data monitoring system, TIM = Transfer ICU and Medication reconciliation program

The study procedures are summarized in Fig. 3.

### The gold standard medication lists

In order to detect MTEs, we defined a gold standard against which the actual prescribed medications are compared.

#### ICU admission

The gold standard is defined as the Best Possible Medication History (BPMH), which includes name, dosage, frequency, and route of all medication taken by a particular patient. The BPMH is created as follows: a pre-admission medication list is collected from the community pharmacy, which is combined with other available information about the patient’s medication, such as a letter from the Emergency department (if applicable), medication used in the general ward or medication lists from the operating room (OR) (if applicable). This medication list is verified by means of an interview with the patient and/or a relative, which will result in a BPMH.

#### ICU discharge

The gold standard is defined as the Best Possible General Ward Medication List 24 h after the ICU discharge (BPML-GW24). The BPML-GW24 includes name, dosage, frequency, and route of all medication that should ideally be prescribed within 24 h after ICU discharge. This list is based on the BPMH, on information found in the PDMS or the HIS of the ICU, on medication prescribed in the CPOE/CDS and, whenever necessary, on interviewing the ward doctor afterwards.

### Collection of medication lists

#### Pre-intervention phase

For each included patient discharged from the ICU for at least 24 h, a pharmacist generates a BPMH and a BPML-GW-24 in order to trace medication transfer errors.

#### Post-intervention phase

The pharmacist generates a BPMH for patients newly admitted to the ICU and the Best Possible ICU Medication Discharge List (BPMDL-ICU) for patients planned to be discharged. One day past ICU discharge, a medication chart of current medication in the CPOE/CDS will be printed and a BPML-GW24 will be compiled.

### Outcome measures

Figure 1 presents a summary of outcome measures.

The primary outcome measures of this study are the proportion of patients with one or more MTEs at admission to the ICU and the proportion of patients with one or more MTEs after discharge from the ICU.

An MTE at admission is defined as an unintentional discrepancy between BPMH and medication prescribed within 24 h after admission to the ICU.

An MTE at discharge is defined as an unintentional discrepancy between the BPML-GW24 and the medication chart of the patient within 24 h after discharge.

**Fig. 4:**
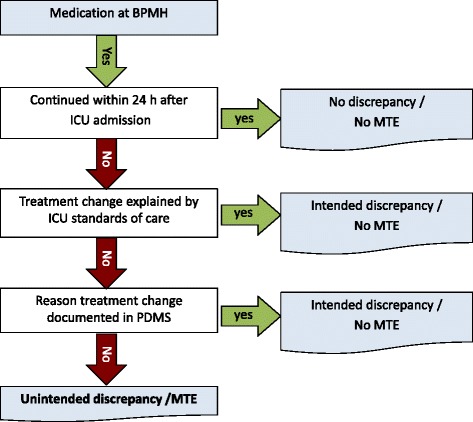
Flow chart. Assessment of unintended medication discrepancies. BPMH = best possible medication history, ICU = intensive care unit, MTE = medication transfer error, PDMS = patient data monitoring system

Whether a discrepancy at admission is unintentional or not is based on information documented in the PDMS, the ICU standards of care and the ICU pharmacist’s interpretation of the situation (see Fig. 4 for the assessment of unintended medication discrepancies).

The secondary outcome measure of this study is the proportion of patients with a potential adverse drug event (pADE) score due to an MTE, both at the moment of admission and at the moment of discharge. In addition, the pADEs scores and the associated costs are secondary outcome measures. A pADE score is defined as a potential and severity score for discomfort, harm and/or clinical deterioration caused by an unintentional discrepancy [19].

Determination of the potential and severity that a patient will experience harm due to an MTE will be based on the methodology described by Nesbit et al [19]. The same method was recently used by Gallagher et al [20]. This method uses the following categories for probability and severity of harm: 0 (zero; no harm expected by the MTE), 0.01 (very low: some harm is expected, but not clinically relevant), 0.1 (low: some harm is expected but poorly clinically relevant), 0.4 (medium; harm is expected, clinically relevant) or 0.6 (high; harm is expected, life threatening). In order to assign the probability and severity category for each MTE, we will present all MTEs at ICU admission or discharge to two assessors; one hospital pharmacist/clinical pharmacologist and one internist/clinical pharmacologist in training, who will, independently from each other, make a judgment based on clinical data of the patient. In case of differences between the judgments, the assessors will come together to reach consensus. If consensus cannot be reached, the case will be presented to a internist/clinical pharmacologist, who will make the final decision.

Next, our preliminary cost-effectiveness analysis will estimate the incremental costs per prevented MTE of the TIM compared to usual care, from the hospital’s perspective. Costs will comprise both the costs incurred by the reconciliation process and cost avoidance due to preventing MTEs and their pADEs.

The cost avoidance of the TIM program will be determined through summation of the individual pADE judgment scores of the MTEs and multiplying it by the cost of an ADE in the pre-intervention period compared to the post-intervention period.

For measuring the cost of an ADE, we will estimate the medical consumption associated with the consequences of harm, such as number of ICU days and medical specialist- and nursing staff time, by means of expert opinion and by using previous literature [7, 9, 19–21]. These will be valued using the reference prices of the ‘Dutch Manual for Costing’ [22]. In case reference prices are not available, medical consumption will be valued with the tariffs provided by the Dutch Healthcare Authority [23]. Costs of potential adverse drug events will be estimated using previous literature [6–8, 10].

Costs incurred by the reconciliation process are restricted to labour. The number of minutes spent per patient by the pharmacist will be measured during the intervention. These minutes will be valued based on standardised costs per minute, which equal the normative income of the pharmacist (collective labour agreement) divided by the number of workable minutes per year [24].

All costs will be based on Euro cost data. If necessary, costs will be adjusted to the year of data collection using the general price index from the Dutch Central Bureau of Statistics [23].

### Data collection

We will screen hospital patient records, PDMS records, CPOE/CDS medication lists, BPMH and BPML-GW24. Collected data from these sources will be categorized into three groups: patient data, medication data and intervention program data. Data will be collected in case report forms (CRFs). All data input in the CRF will be cross-checked by two pharmacists.

#### Patient data

The following patient data are extracted from the medical records: age, gender, APACHE II (Acute Physiology and Chronic Health evaluation, score 0–74), SAPS II (Simplified Acute Physiology Score, scores varying from 0 to 163 and a predicted mortality rate between 0 and 100%)), reason for the ICU admission, deceased during the ICU stay, length of ICU stay, specialty of referring physician, surgical patient or non-surgical patient, ward from which the patient was referred to the ICU [emergency room (ER), general ward, OR, cardiac care unit (CCU), home] and type of admission [planned, emergency, organ transplantation]. Both APACHE II and SAPS II are general illness severity scores, APACHE is the world most widely applied severity of illness score, whereas SAPS is developed in France and validated in 12 European/North American countries. Both scores are based on the worst values recorded in the first 24 h of the ICU stay and are used in the ICU to predict outcome. They characterize disease severity and degree of organ dysfunction [25].

#### Medication data

The following information is collected for all medication on the BPMH and/or BPML-GW-24: name of medicine, dose form, medication group, dose and frequency.

We will register for every BPMH medication whether it is (1) prescribed in the PDMS within 24 h after admission, (2) registered in the PDMS as “pre-admission medication”, (3) prescribed pre-ICU in CPOE/CDS and (4) registered as “admission medication” in the ICU discharge letter.

We will register for every BPML-GW24 medication whether it is (1) prescribed in the CPOE/CDS within 24 h after the ICU discharge, (2) in use at discharge on the ICU according tot the PDMS medication chart, (3) noted on the PDMS discharge letter as “ICU medication”.

All discrepancies are scored as intended or non-intended and finally the discrepancy type: omission, start, stop, different dose, substitution or incorrect prescribing will be scored.

#### Intervention program data

During the post-intervention phase the process of medication reconciliation is qualified as A, B or C. Quality A is defined as an optimal reconciliation based on a recent, reliable community pharmacy medication list and a reliable verification with patient and/or his representative. Quality B is defined as an intermediate reconciliation; the pharmacy record is recent and reliable and screened for obvious errors, verification with patient or relative is however not reliable. And finally quality C is defined as a sub-optimal reconciliation with either the community pharmacy medication list lacking or the patient/representative verification lacking.

Other data collected on medication reconciliation are: date of reconciliation, within 24 h of the ICU stay or not, verification with patient and/or representative, time spent on verification (patient’s interview), the total reconciliation time and finally the number of medications on the BPMH and BPML-GW24.

### Data analysis

#### Sample size

The primary outcome of this study is the proportion of patients with one or more transfer errors at admission to the ICU and at discharge from the ICU.

Based on literature the expected proportion of patients with medication errors due to transfer between wards within one hospital is 62% [18]. Based on a conservative interpretation of this study, it was estimated that this proportion is 30% in our study. With an estimated 50% reduction of errors due to the intervention, an alpha of 0.05 and a power of 0.80 the calculated sample size is 133. This sample size is based on patients being alive at discharge for both the pre-intervention as the post-intervention phase.

With an estimated mortality of 35%, in each measurement phase 205 patients should be included. Based on 1500 (Haga Teaching Hospital) and 1800 (Erasmus MC) admissions a year, an estimated loss of 30% due to the ICU stay less than 24 h and another 35% loss due to weekend ICU stay, we estimate a study period of 7 weeks for Erasmus MC and 8 weeks for Haga. To be on the safe side and to measure during a robust intervention period, we will double the number of calculated weeks. Therefore a pre- and post-intervention period of 14 weeks is chosen.

#### Statistical analysis

All data will be entered in MS Access 2007 and will be analyzed with IBM SPSS Statistics 24.

Patients from the intervention and control group will be compared for all baseline characteristics using *t*-test for continuous normally distributed variables, Mann-Whitney *U*-test for continuous non-normally distributed variables and chi square test for categorical variables.

For the primary outcome (MTEs) relative risks and 95% confidence intervals will be calculated. As the study has a pre- and post-design, results will be corrected for potential confounders by using multivariate logistic regression analysis.

#### Subgroup analyses

Since the usual care provided in both hospitals is not completely the same, the usual care may have impact on the primary and secondary outcomes, i.e. the number of MTE and the risk of ADEs. Therefore we will assess in a secondary analysis, whether the effect of the intervention is modified by hospital site and we will present subgroup analyses per site.

## Discussion

The aim of this study is to determine the effect of the TIM program on the proportion of patients with medication transfer errors (MTEs) at admission to and at discharge from the ICU, and on the number, severity and cost of potential adverse drug events (pADEs). Although a randomized controlled trial is generally considered to be the standard for comparing interventions, unfortunately a randomized design is not feasible for this particular investigation. Previous experience with similar projects has demonstrated that usual care is strongly influenced by an intervention program like TIM, as doctors and other healthcare providers are likely to learn from the program. Doctors will gain awareness of problems associated with transfers and may consequently change their prescribing behavior and the organization of care.

To be able to compare the effects of TIM with usual care while avoiding a learning effect, we chose a before and after design. Although this is conceived to be a weaker design, we expect this study to have several strengths. First, pharmacists’ knowledge of the ICU is combined with pharmacists’ knowledge and skills regarding medication transfer and medication reconciliation. In both hospitals, dedicated ICU pharmacists participated in designing the TIM program. These ICU pharmacists have a thorough understanding of the ICU work processes, the ICU medication issues and the ICU culture. Their medication transfer and reconciliation skills have been developed in the previous years during several pilots and studies on medication transfer and reconciliation in different settings (admission and discharge from the hospital) [26–28]. Combining all these competencies has led to a robust design that is tailor-made for both hospitals.

A second strength is that ICU doctors participated in the final design of the TIM intervention. The TIM study design was tested from an ICU-doctor perspective during a study design meeting with 4 internist-intensivists, 2 from each hospital. This led to some adaptations in the TIM program and in the outcome measures.

Thirdly, the TIM intervention will be performed by ICU pharmacists, which will make the cooperation between pharmacists and the ICU doctors more effective.

The fourth strength of the study is that it is performed in two different settings, a general teaching hospital and a university medical center setting, which will provide stronger results compared to a single site setting.

This study has a number of limitations. First, this program is performed during workdays but not during weekends. This may lead to selection bias because quality of care can be more compromised in weekends than on weekdays due to week/weekend shifts and limited staff in weekends on the general wards. Second, the study does not include a hospital setting in which the PDMS and the CPOE/CDS system are part of the HIS. An unknown part of our MTEs can be due to transcription problems between different prescribing systems, which would not occur in hospitals which only have one prescribing system as part of the HIS. However, even if only one prescribing system is used throughout the ICU admission and discharge, the doctor of the general ward may omit restarting pre-admission medication after ICU discharge. Be that as it may, this study will not be able to answer questions about the effect of using two different prescribing systems as such. Third, although this study will be performed in two different settings, results would be even stronger and more generalizable if more hospitals were included.

Fourth, the study will measure *potential* adverse drug events, assessed by a multidisciplinary panel, rather than *actual* adverse events. Therefore, in case of positive results on the primary outcome, future studies will be needed to study the effect on actual patient harm.

Finally, a period of 14 weeks for the post-intervention measurement is relatively short; longer periods may be necessary in order to determine whether the results remain consistent over time.

While most studies focus on the effect of medication reconciliation programs at the moment of hospital admission or discharge, this study focuses on ICU transfers and will be able to evaluate the clinical and financial impact of a comprehensive program on continuity of care. The findings of this study will provide health care professionals as well as patients, health care managers and policy makers with valuable information on a crucial safety intervention in the ICU.

## Abbreviations

APACHE II: Acute physiology and chronic health evaluation II
BPMDL-ICU: Best possible ICU medication discharge list
BPMH: Best possible medication history
BPML-GW24: Best possible general ward medication list 24 hours after ICU discharge
CCU: Cardiac care unit
CPOE/CDS system: Computerized physician order entry systems with clinical decision support
CRF: Case report form
ER: Emergency room
HIS: Hospital information system
ICU: Intensive care unit
METC: Medical ethics committee
MTE: Medication transfer error
OR: Operating room
pADE: *probable* adverse drug event
PDMS: Patient data monitoring system
SAPS II: Simplified acute physiology score
TIM: Transfer ICU and medication reconciliation program
